# Prediction of lymph node metastasis in advanced gastric adenocarcinoma based on dual-energy CT radiomics: focus on the features of lymph nodes with a short axis diameter ≥6 mm

**DOI:** 10.3389/fonc.2024.1369051

**Published:** 2024-03-01

**Authors:** Yang You, Yan Wang, Xianbo Yu, Fengxiao Gao, Min Li, Yang Li, Xiangming Wang, Litao Jia, Gaofeng Shi, Li Yang

**Affiliations:** ^1^ Department of Computed Tomography and Magnetic Resonance, The Fourth Hospital of Hebei Medical University, Shijiazhuang, China; ^2^ CT Collaboration, Siemens Healthineers Ltd., Beijing, China; ^3^ Department of Computed Tomography and Magnetic Resonance, Xing Tai People’s Hospital, Xingtai, China

**Keywords:** advanced gastric cancer, lymph node metastases, radiomics, dual-energy, CT

## Abstract

**Objective:**

To explore the value of the features of lymph nodes (LNs) with a short-axis diameter ≥6 mm in predicting lymph node metastasis (LNM) in advanced gastric adenocarcinoma (GAC) based on dual-energy CT (DECT) radiomics.

**Materials and methods:**

Data of patients with GAC who underwent radical gastrectomy and LN dissection were retrospectively analyzed. To ensure the correspondence between imaging and pathology, metastatic LNs were only selected from patients with pN3, nonmetastatic LNs were selected from patients with pN0, and the short-axis diameters of the enrolled LNs were all ≥6 mm. The traditional features of LNs were recorded, including short-axis diameter, long-axis diameter, long-to-short-axis ratio, position, shape, density, edge, and the degree of enhancement; univariate and multivariate logistic regression analyses were used to establish a clinical model. Radiomics features at the maximum level of LNs were extracted in venous phase equivalent 120 kV linear fusion images and iodine maps. Intraclass correlation coefficients and the Boruta algorithm were used to screen significant features, and random forest was used to build a radiomics model. To construct a combined model, we included the traditional features with statistical significance in univariate analysis and radiomics scores (Rad-score) in multivariate logistic regression analysis. Receiver operating curve (ROC) curves and the DeLong test were used to evaluate and compare the diagnostic performance of the models. Decision curve analysis (DCA) was used to evaluate the clinical benefits of the models.

**Results:**

This study included 114 metastatic LNs from 36 pN3 cases and 65 nonmetastatic LNs from 28 pN0 cases. The samples were divided into a training set (n=125) and a validation set (n=54) at a ratio of 7:3. Long-axis diameter and LN shape were independent predictors of LNM and were used to establish the clinical model; 27 screened radiomics features were used to build the radiomics model. LN shape and Rad-score were independent predictors of LNM and were used to construct the combined model. Both the radiomics model (area under the curve [AUC] of 0.986 and 0.984) and the combined model (AUC of 0.970 and 0.977) outperformed the clinical model (AUC of 0.772 and 0.820) in predicting LNM in both the training and validation sets. DCA showed superior clinical benefits from radiomics and combined models.

**Conclusion:**

The models based on DECT LN radiomics features or combined traditional features have high diagnostic performance in determining the nature of each LN with a short-axis diameter of ≥6 mm in advanced GAC.

## 1 Introduction

Gastric cancer (GC) is the fourth leading cause of cancer-related death worldwide (1) and the third leading cause of cancer-related death in China (2, 3). Lymph node (LN) metastasis (LNM) is the main metastatic pathway of GC and an independent predictor of patient prognosis (4–6). Accurate preoperative judgment of LN status has guiding significance for selecting treatment options and evaluating patient prognosis (7–9). GC has abundant lymphatic drainage and skip metastasis, making it difficult to determine LNM before surgery accurately. Computed tomography (CT) is the main imaging method for the preoperative assessment of LNM in GC. However, there is no uniform standard for judging LNM, and the overall accuracy rate is only approximately 60% (10, 11).

Radiomics mines massive quantitative features from image data, which can reflect the spatial distribution of voxels and better reflect tumor heterogeneity (12, 13). Radiomics has been shown to predict malignant LNM (13–16). Compared to traditional CT data, dual-energy CT (DECT) data can enrich the radiologic characteristics and provide more valuable characteristics for judging LNM. Most previous studies have investigated the radiomics features of the primary tumor, which can only predict the presence or absence of LNM (17–19), and the nature of each LN and N stage cannot be accurately determined. Few studies on LNM prediction in GC based on LN radiomics features exist. Patients with GC are more likely than healthy people, and patients with advanced GC are more likely than those with early GC to have perigastric LNs with a short-axis diameter ≥ 6 mm (20, 21). Currently, there is no uniform standard for judging LNM. It is generally believed that the larger the LN, the more likely it is to metastasize, and it is difficult to characterize LNs with a short diameter of 6–10 mm in clinical work. Therefore, this study took perigastric LNs with a short-axis diameter of ≥6 mm as the research object to investigate the value of DECT LN radiomics features in predicting LNM in advanced gastric adenocarcinoma (GAC).

## 2 Materials and methods

### 2.1 Research object

**Figure 1:**
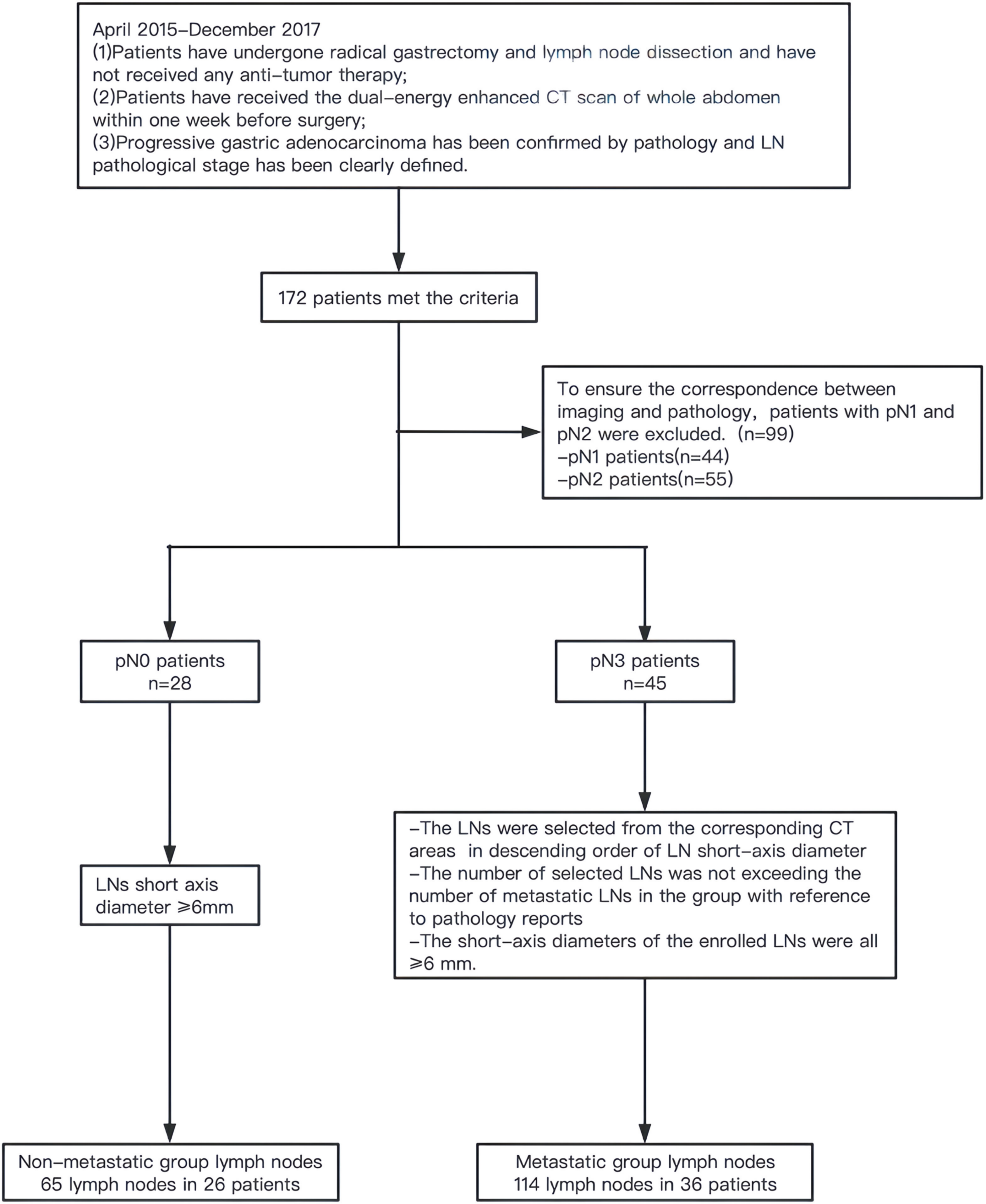
Flow chart of lymph node enrollment in the metastatic group and nonmetastatic group.

This study was approved by the Ethics Committee of the Fourth Hospital of Hebei Medical University. Data from patients with GC undergoing surgery at our hospital from April 2015 to December 2017 were retrospectively analyzed. Inclusion criteria: (1) patients received radical gastrectomy and LN dissection and had not received antitumor therapy before surgery; (2) whole abdominal dual-energy enhanced CT scan was performed within 1 week before surgery; and (3) postoperative pathologically confirmed advanced GAC wherein LN pathological staging was clear. A total of 172 patients met the above criteria, including 28 pN0 cases, 44 pN1 cases, 55 pN2 cases, and 45 pN3 cases. As this was a retrospective study, achieving the exact correspondence between imaging and pathology was difficult. To ensure maximum correspondence, metastatic LNs were only selected from pN3 cases, and nonmetastatic LNs were selected from pN0 cases. Based on the pathology report, the LNs were selected from the corresponding CT areas of pN3 cases in a descending order of LN short-axis diameter in the metastatic group. The selected number of LNs was not greater than the number of metastatic LNs in this group, and the diameter of the short axis was ≥6 mm. In the nonmetastatic group, LNs with short-axis diameters ≥6 mm were selected from pN0 cases (**Figure 1**).

### 2.2 Dual-energy CT imaging

Examination methods and parameters: Patients underwent 6–8 h of fasting before examination, were intramuscularly injected with 10 mg of anisodamine 10 min before scanning, and were orally given 6 g of gas-producing powder or 800–1000 mL of warm water to fill the stomach cavity. Second-generation dual-source CT (Somatom Definition Flash, Siemens Healthcare, Erlangen, Germany) was used for scanning. The scanning range was from the top of the diaphragm to the level of the lower border of the pubic symphysis. For the plain scan, the tube voltage was 120 kV, the tube current was 210 mAs, the collimator width was 128 × 0.6 mm, and the pitch was 0.9. Iohexol (300 mgI/mL) was injected through the cubital vein at a rate of 3.0 mL/s using a high-pressure injector, with a dose of 1.5 mL/kg body weight, and dual-energy dual-phase scanning was performed at 25 s and 70 s after injection.

The scanning parameters were as follows: The voltages of tubes A and B were 100 kV and Sn 140 kV, respectively. Care Dose 4D was turned on, the reference tube currents were 230 mAs and 178 mAs, respectively, collimator width was 32 × 0.6 mm, pitch was 0.55, slice thickness was 5.0 mm, and increment was 5.0 mm. The venous phase data were reconstructed into equivalent 120 kV linear fusion images (fusion coefficient of 0.5) and iodine maps (IM), with a reconstruction thickness of 1.0 mm and an increment of 1.0 mm.

### 2.3 Building the traditional features model

Two radiologists with 5 years (observer 1) and 17 years (observer 2) of experience in abdominal imaging diagnosis independently analyzed traditional features in venous phase fusion images without knowing the postoperative pathology. When their opinions differed, they reached an agreement through consultation. LN traditional features included: short axis diameter, long axis diameter, long-to-short axis ratio (LSR), position (D1 station; non-D1 station), shape (nearly round shape, LSR ≤1.5; non-round shape, LSR >1.5), density (homogeneous; heterogeneous), edge (regular; unregular), and the degree of enhancement (mild enhancement, <20 HU; moderate to strong enhancement, ≥20 HU). Based on the training set data, univariate logistic regression analysis was used to screen out traditional features related to LNM, and then backward stepwise multivariate logistic regression analysis was performed to establish the traditional features model.

### 2.4 Building the radiomics model

#### 2.4.1 Image segmentation

Using Radiomics software (Frontier, Siemens Healthcare), in the venous phase fusion image, observer 1 manually delineated a two-dimensional region of interest (ROI) at the maximum level of the LN along the inner edge of the LN contour, which was reviewed by observer 2. Disagreement was resolved through negotiation, and thereafter, the ROI was copied on the IM using the software to ensure consistency in the delineation.

#### 2.4.2 Feature extraction, screening, and model building

**Figure 2:**
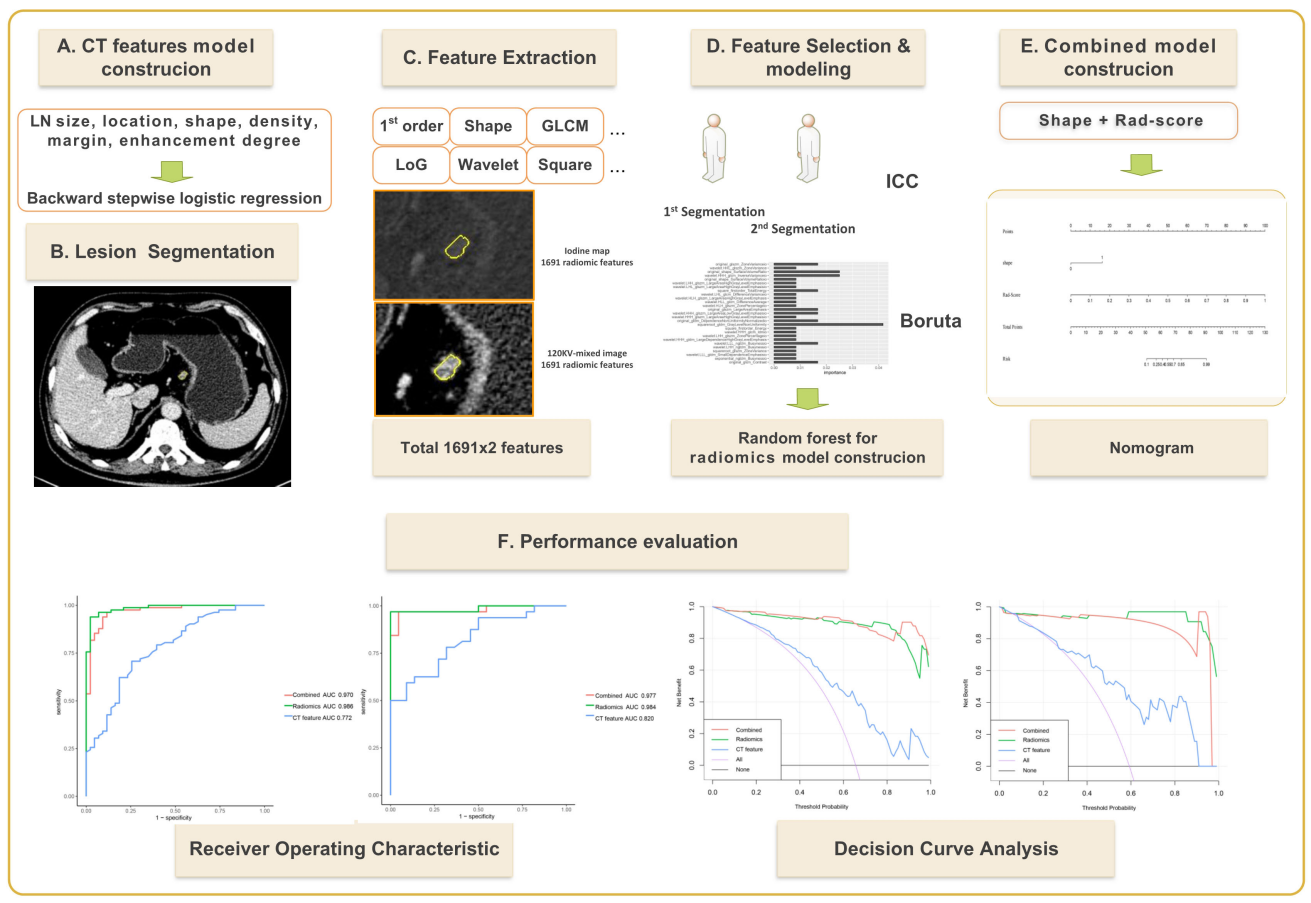
Flow chart for establishing a dual-energy computed tomography-based model for predicting lymph node metastasis of lymph nodes with a short-axis diameter ≥6 mm in gastric adenocarcinoma.

Radiomics software was used to extract radiomics features for each ROI in the training set of venous phase fusion images and IM using Radiomics software. Feature screening method: (1) In total, 50 cases were randomly selected, observer 1 performed the second delineation after a 1-month interval, and the radiomics features with an intra-class correlation coefficient (ICC) >0.8 were retained. (2) The features were further screened using the Boruta, an algorithm to screen important features by comparing their importance with that of randomly generated shadow features. Because of its high computational efficiency, it is suitable for datasets with many variables (22). In this study, Boruta feature selection was implemented through the R package *Boruta* (Version 7.0.0), and the selected key features were used to establish radiomics models. The radiomics workflow is shown in **Figure 2**.

### 2.5 Building the combined model

The traditional features (*P*<0.05) with statistical significance in the univariate logistic regression analysis and the radiomics score (Rad-score) based on the radiomics model were subjected to multivariate logistic regression analysis, and the independent predictors of LNM were screened to construct the combined model and nomograms.

### 2.6 Statistical methods

R software 4.0.5 (http://www.Rproject.org) was used. Count data were compared with the χ^2^ test or Fisher’s exact test. Measurement data conforming to a normal distribution are expressed as the mean ± standard deviation, and an independent samples t-test was used to compare the two groups. Measurement data that do not conform to a normal distribution are expressed as the median [upper and lower quartiles], and the Mann−Whitney U test was used to compare the two groups. *P*<0.05 was considered statistically significant. Receiver operating characteristic (ROC) curves were used to evaluate the diagnostic performance of the models, and the DeLong test was used to compare the differences in diagnostic performance among the models. Decision curve analysis (DCA) was used to evaluate the clinical benefit of the model.

## 3 Results

### 3.1 Comparison of the baseline features between the training and validation sets

A total of 36 pN3 cases with 114 LNs were included in the metastatic group: 27 males and 9 females, with an average age of 56.7 ± 10.2 years. A total of 26 pN0 cases with 65 LNs were included in the nonmetastatic group: 22 males and 4 females, with an average age of 60.7 ± 9.1 years. The two groups of LNs were randomly assigned to the training set (n=125) and the validation set (n=54) in a 7:3 ratio. There were no significant differences in clinical and traditional features between the training and validation sets (all *P*>0.05) (**Table 1**).

**Table 1:** Comparison of clinical and traditional features of the training and validation sets.

Features	Training cohort (n=125)	Validation cohort (n=54)	*P value*
**Age, M[*Q*_25_;*Q*_75_]**	58.0 [56.0;64.0]	58.0 [56.0;63.8]	0.908
**Sex, No. (%)**			0.872
Female	18 (14.4%)	9 (16.7%)	
Male	107 (85.6%)	45 (83.3%)	
Size, M[*Q*_25_;*Q*_75_]			
Short axis diameter	0.79 [0.67;0.98]	0.84 [0.66;1.05]	0.663
Long axis diameter	1.12 [0.91;1.52]	1.20 [0.92;1.61]	0.448
Long-to-short axis ratio	1.35 [1.20;1.57]	1.30 [1.21;1.59]	0.929
**Location, No. (%)**			0.494
D1 station	100 (80.0%)	40 (74.1%)	
Non-D1 station	25 (20.0%)	14 (25.9%)	
**Shape, No. (%)**			0.917
Nearly round shape	97 (77.6%)	43 (79.6%)	
Non-round shape	28 (22.4%)	11 (20.4%)	
**Density, No. (%)**			0.189
Homogeneous	119 (95.2%)	48 (88.9%)	
Heterogeneous	6 (4.80%)	6 (11.1%)	
**Edge, No. (%)**			1.000
Regular	63 (50.4%)	27 (50.0%)	
Irregular	62 (49.6%)	27 (50.0%)	
**Degree of enhancement, No.(%)**			0.749
Mild	88 (70.4%)	40 (74.1%)	
Moderate to strong	37 (29.6%)	14 (25.9%)	

### 3.2 The traditional feature model

Univariate logistic regression analysis showed that the LN short-axis diameter, long-axis diameter, shape, and edge were correlated with LNM (all *P*<0.05). Backward stepwise multivariate logistic regression analysis showed that LN long axis diameter and shape were independent predictors of LNM (all *P*<0.05) (**Table 2**). Based on this, the traditional features model was built, and its area under the curve (AUC), accuracy, sensitivity, and specificity in the training set and validation set were 0.772, 0.720, 0.707, 0.744 and 0.820, 0.741, 0.781, 0.682, respectively.

**Table 2:** Univariate and multivariate logistic analysis of traditional features on lymph node metastasis in gastric adenocarcinoma.

Features	Univariate			Multivariate		
	beta	OR (95%CI)	*P value*	beta	OR (95%CI)	*P value*
Short axis diameter	4.382	79.999 (10.046–1079.203)	<0.001	-	-	-
Long axis diameter	1.475	4.369 (1.698–13.035)	0.004	2.808	16.585 (4.318–86.079)	<0.001
Long-to-short axis ratio	-0.984	0.374 (0.111–1.224)	0.105	-	-	-
Location	0.513	1.670 (0.672–4.084)	0.261	-	-	-
Shape	1.240	3.457 (1.459–8.418)	0.005	2.429	11.352 (3.635–41.923)	<0.001
Density	1.003	2.727 (0.422–53.168)	0.367	-	-	-
Edge	1.232	3.427 (1.588–7.717)	0.002	-	-	-
Degree of enhancement	-0.214	0.808 (0.365–1.821)	0.600	-	-	-

OR, odds ratio; CI, confidence interval.

### 3.3 The radiomics models

**Figure 3:**
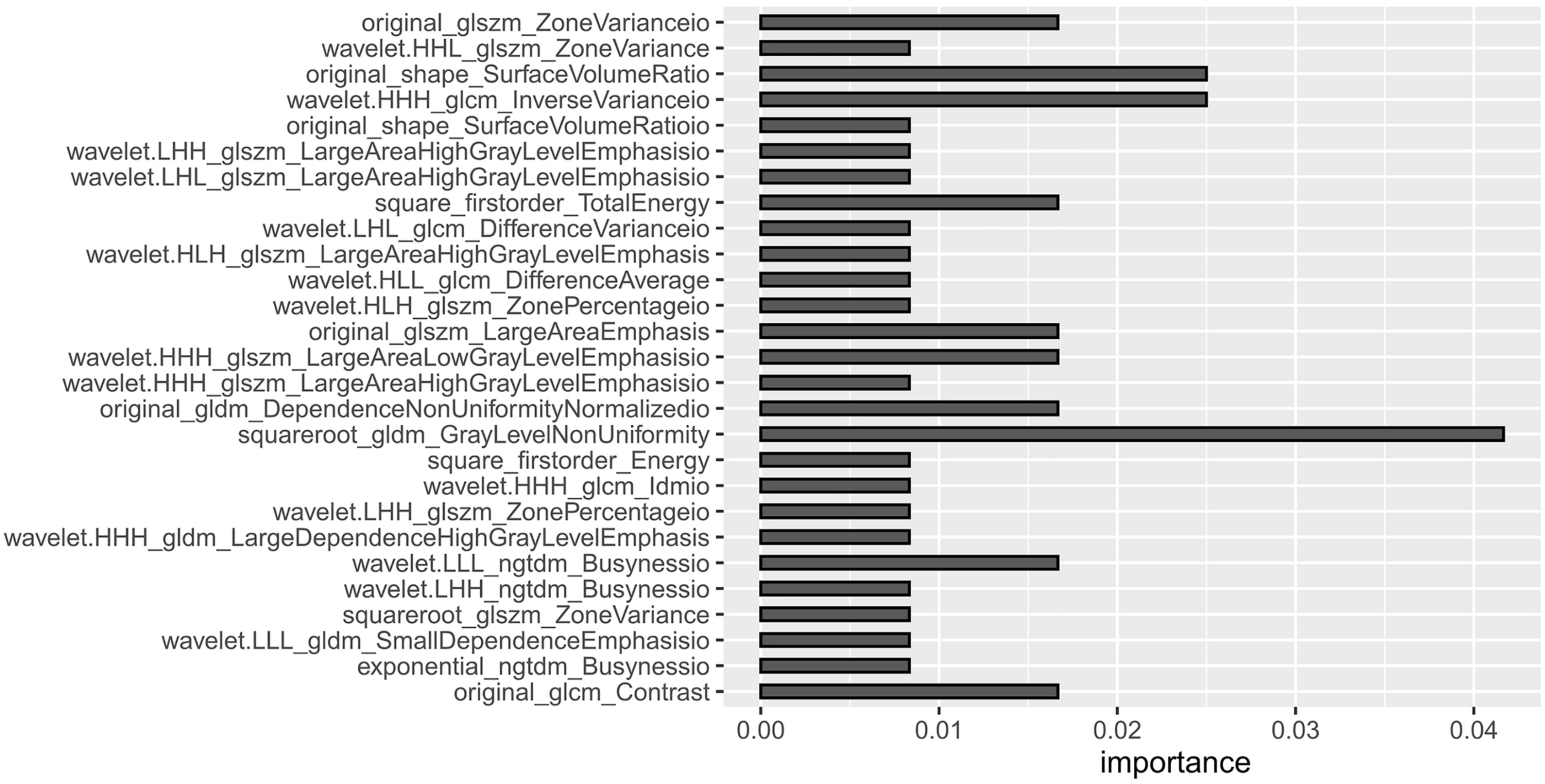
A total of 27 radiomics features in the radiomics model.

**Figure 4:**
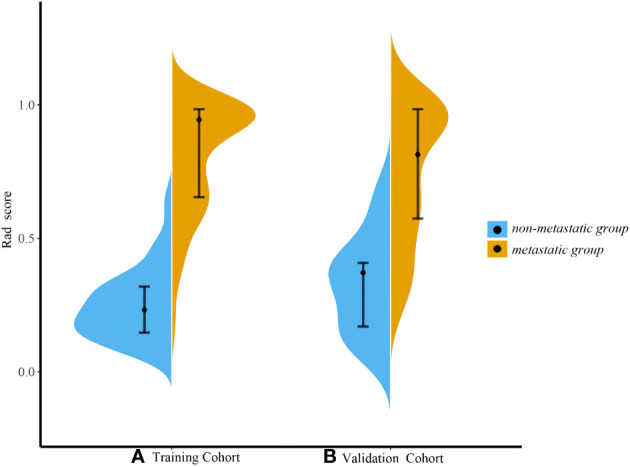
Violin plot of Rad-scores. **(A)** Training set **(B)** Validation set. The violin plot shows the distribution of the overall Rad-score. The wider shape represents a higher probability of the same Rad-score, and a narrower shape represents a lower probability of the same Rad-score.

Based on the venous phase fusion image and IM, 1691 radiomics features were extracted from each; thus, 3382 radiomics features were extracted, including three types: 26 shape features, 19 first-order features, and 75 texture features. Texture features included 24 gray level co-occurrence matrices (GLCM), 16 gray level run length matrices (GLRLM), 16 gray level size zone matrices (GLSZM), 14 gray-level dependence matrices (GLDM) and 5 neighboring gray tone difference matrices (NGTDM). After the ICC test and Boruta algorithm screening, 27 radiomics features were finally retained for model building, of which 16 features were from IM and 11 were from venous phase fusion images (**Figure 3**). The Rad-score of the metastatic group was higher than that of the nonmetastatic group (*P*<0.01) (**Figure 4**). The AUC, accuracy, sensitivity, and specificity of the radiomics model in the training and validation sets were 0.986, 0.952, 0.939, and 0.977 and 0.984, 0.963, 0.969, and 0.955, respectively. Delong’s test showed no significant difference in the AUC of the radiomics model between the training and validation sets (*P*=0.948).

### 3.4 The combined model

Multivariate logistic regression analysis showed that LN shape and Rad-score were independent predictors of LNM (all *P*<0.05). Based on this, the combined model was constructed, and the AUC, accuracy, sensitivity, and specificity of the training set and validation set were 0.970, 0.936, 0.963, and 0.884, and 0.977, 0.870, 0.969, and 0.727, respectively.

### 3.5 Comparison of the three models

**Figure 5:**
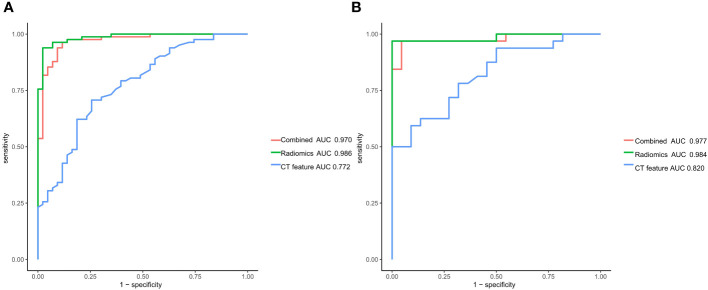
Receiver operating characteristic curves of the traditional features model, radiomics model and combined model for predicting lymph node metastasis in gastric cancer. **(A)** Training set **(B)** Validation set.

**Figure 6:**
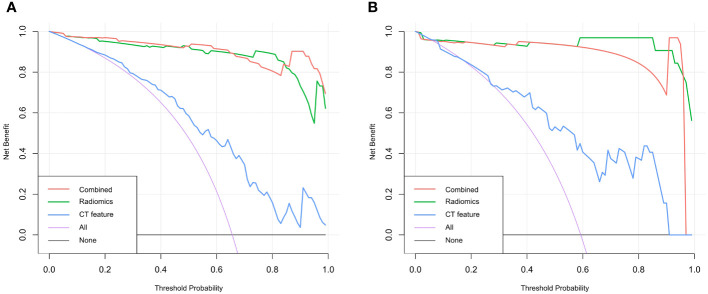
Decision curve analysis curves of the traditional features, radiomics model and combined model for predicting lymph node metastasis in gastric cancer. **(A)** Training set **(B)** Validation set.

**Figure 7:**
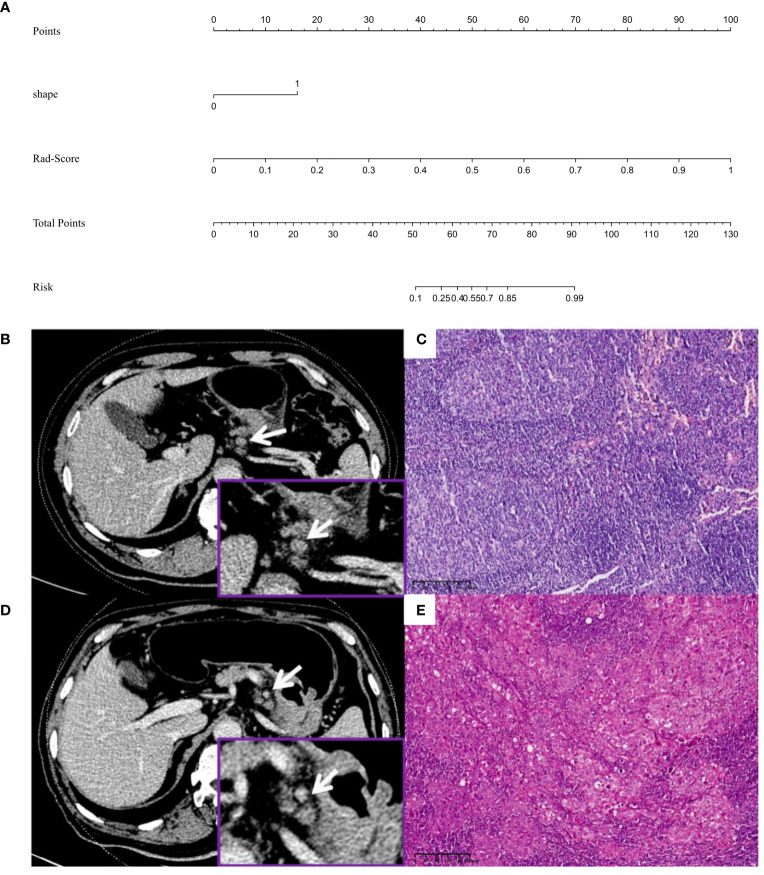
**(A)** Nomogram for predicting lymph node metastasis in advanced gastric adenocarcinoma patients with a short-axis diameter of ≥6 mm. **(B, C)** Male, 58 years old, GAC b: A No. 3 group LN (arrow), which was nearly round and approximately 11.1×9.0 mm, was diagnosed by the traditional features model as metastatic LN (prediction score was 0.742, cutoff value was 0.638), diagnosed by the radiomics model as nonmetastatic LN (Rad-score was 0.137, cutoff value was 0.401), diagnosed by the combined model as nonmetastatic LN (predictive score was 0.001, cutoff value was 0.614) c: Pathology confirmed it as nonmetastatic LN (HE, 10×), and the pathological stage was T4aN0M0. **(D, E)** Male, 71 years old, GAC D: A No.3 group LN (arrow), which was nearly round and approximately 8.0×7.3 mm, was diagnosed as nonmetastatic LN by the traditional features model (prediction score was 0.546, cutoff score was 0.638), diagnosed by the radiomics model as metastatic LN (Rad-score was 1.000, cutoff value was 0.401), diagnosed by the combined model as metastatic LN (prediction score was 1.000, cutoff value was 0.614) e: Pathology confirmed it as metastatic LN (HE, 10×), and the pathological stage was T4aN3M0.

The DeLong test was used to compare the AUCs of the traditional features, radiomics and combined models. The AUCs of the training and validation sets in the radiomics and combined models were higher than those of the traditional features model (all *P*<0.01). There was no significant difference in AUCs between the radiomics model and the combined model (all *P*>0.0167) (**Table 3**, **Figure 5**). The DCA curves of the three models were higher than the two reference lines, and the net clinical benefit of the radiomics model and the combined model was higher than that of the traditional features model (**Figure 6**). The nomogram of the combined model is shown in **Figure 7**.

**Table 3:** Comparison of AUCs of the traditional features model, radiomics model and combined model.

Cohorts	Area under the curve (95% confidence interval)			*P* value (0 vs 1)	*P* value (0 vs 2)	*P* value (1 vs 2)
	Traditional features model (0)	Radiomics model (1)	Combined model (2)			
Training cohort	0.772	0.986	0.970	<0.001^*^	<0.001^*^	0.027
	(0.687–0.858)	(0.970–1.000)	(0.942–0.998)			
Validation cohort	0.820	0.984	0.977	0.005^*^	0.007^*^	0.275
	(0.711–0.930)	(0.953–1.000)	(0.941–1.000)			

^*^ *P*<0.0167.

## 4 Discussion

Accurate preoperative judgment of LNM in GC is crucial for selecting treatment options and determining the extent of LN dissection. In this study, both the radiomics model established based on the radiomics features of DECT LNs and the combined model based on the traditional features of LNs and the radiomics features of DECT LNs have high diagnostic performance. The models can accurately determine the nature of LNs with a short-axis diameter of ≥6 mm in advanced GAC before surgery. This has important clinical value in the preoperative judgment of N staging, individualized treatment plans, and patient prognosis.

This study found that the short-axis diameter, long-axis diameter, shape, and edge of perigastric LNs were associated with LNM in advanced GAC, and long-axis diameter and shape were independent predictors of LNM. Unlike previous research results (23, 24), the degree of LN enhancement did not become an independent predictor of LNM, which may be related to the larger LNs with a short-axis diameter ≥6 mm included in this study. The enlargement of LNs in the nonmetastatic group may be due to inflammatory stimulation or reactive hyperplasia. This resulted in increased blood supply to the LNs, thereby narrowing the difference in the degree of enhancement between the metastatic and nonmetastatic LNs in this study.

By extracting high-throughput quantitative features, radiomics analysis can effectively assess the spatial distribution of voxels that was viewed as highly related to tissue heterogeneity (25, 26). Currently, most relevant studies are based on the radiomics features of primary GC to predict LNM (AUC: 0.704–0.837) (17–19). Individual LNs cannot be independently predicted. Therefore, preoperative LN staging cannot be performed accurately. Yang et al. (27) established models to predict LNM in GC based on the radiomics features of primary tumors and LNs, and the results showed that the performance of the LNs model was better than that of the primary tumor model (AUC: 0.911 vs 0.852), suggesting that the radiomics features of LNs could provide more heterogeneity information of LNs, and could predict LNM more directly and accurately. To avoid the autocorrelation between the features of the primary tumor and LN, and because all the patients had advanced gastric cancer, this study only focused on the features of LNs. In this study, the LNM model for predicting LNM in GC established based on the radiomics features of LNs had AUCs of 0.986 and 0.984 in the training set and validation set, respectively, with high sensitivity and specificity and good robustness. The diagnostic efficacy was also better than that of the LNM prediction model, combining radiomics features of the primary tumor and LNs of GC reported in the literature (AUC=0.908) (28).

DECT-derived IMs can quantify iodine distribution related to the blood supply, and radiomics analysis based on IM can reflect perfusion-related blood supply heterogeneity through high-throughput quantitative features (24, 29). In this study, radiomic features were extracted from DECT venous phase fusion images and IM, and 27 radiomic features with high correlation with LNM in GC, 16 of which were from IM, were screened. The results suggest that the dual-energy data enriches the radiomics features and provides more valuable features for determining LNM. A total of 23 of the 27 radiomics features were texture features, of which 16 were processed and decomposed by wavelet filters to explore spatial heterogeneity at multiple scales (30), which can objectively reflect the internal details and heterogeneity of LNs and is an important feature for judging the nature of LNs. “square root gldm Gray Level Non-Uniformity” and “wavelet HHH glcm Inverse Variance io” were the two texture features with larger weights.

The diagnostic performance of the combined model established by combining LN shape and Rad-score was comparable to that of the radiomics model, and both were higher than that of the traditional features model, which also confirmed the important value of radiomics features in predicting LNM in advanced GAC.

Our study has the following limitations: 1. This study was a single-center retrospective analysis. The model performance needs to be further verified by a large-sample, multicenter prospective study. 2. Using two-dimensional image segmentation instead of whole tumor segmentation may not reflect all the features of LNs. 3. We focused on venous phase images only, while arterial phase images may provide additional information.

## Data availability statement

The raw data supporting the conclusions of this article will be made available by the authors, without undue reservation.

## Ethics statement

The studies involving humans were approved by the Ethics Committee of the Fourth Hospital of Hebei Medical University. The studies were conducted in accordance with the local legislation and institutional requirements. The ethics committee/institutional review board waived the requirement of written informed consent for participation from the participants or the participants’ legal guardians/next of kin because This study was approved by the Institutional Review Board of the Fourth Hospital of Hebei Medical University, and written informed consent was waived due to the retrospective nature. Written informed consent was not obtained from the individual(s) for the publication of any potentially identifiable images or data included in this article because This study was approved by the Institutional Review Board of the Fourth Hospital of Hebei Medical University, and written informed consent was waived due to the retrospective nature.

## Author contributions

YY: Writing – original draft. YW: Writing – original draft. XY: Writing – original draft. FG: Writing – original draft. ML: Writing – original draft. YL: Writing – original draft. XW: Writing – original draft. LJ: Writing – original draft. GS: Writing – original draft, Writing – review & editing. LY: Writing – original draft, Writing – review & editing.
